# Asymptomatic Hypocalcemia Related to Denosumab Administration in Bone-Metastatic Patient Affected by Colorectal Cancer: A Case Report

**DOI:** 10.2174/0118715303347379241107095529

**Published:** 2024-12-05

**Authors:** Andrea Corsello, Francesco Torino, Annapina De Rosa, Domenica D’Addario, Celestina Cannarsa, Rosa Maria Paragliola

**Affiliations:** 1 Unit of Endocrine Surgery, Ospedale Isola Tiberina - Gemelli Isola, Rome, Italy;; 2 Department of Systems Medicine, Medical Oncology Unit, University of Rome “Tor Vergata”, Rome, Italy;; 3 Endocrinologia Specialistica Ambulatoriale, Azienda Sanitaria Regionale Molise, Termoli, Italy;; 4 Oncologia, Ospedale S. Timoteo, Azienda Sanitaria Regionale Molise, Termoli, Italy;; 5 Medicina di base, Specialistica e Riabilitativa, Azienda Sanitaria Regionale Molise, Termoli, Italy;; 6 Unicamillus-Saint Camillus International University of Health Sciences, Rome, Italy

**Keywords:** Denosumab, hypocalcemia, cancer, bone metastases, osteoporosis, colorectal cancer

## Abstract

**Background:**

Denosumab, a fully humanized monoclonal neutralizing antibody inhibiting the RANK/RANKL/OPG signaling pathway, is widely used for treating patients with bone metastases. However, its use in cancer patients with bone metastases is burdened by the risk of all grades of hypocalcemia, with the severe grade being rare. In the literature, several cases of severe symptomatic hypocalcemia have been reported, particularly in patients with breast and prostate cancers. In this report, we present a rare case of asymptomatic hypocalcemia in a 78 years-old patient with sigmoid cancer and bone metastases.

**Case Presentation:**

Hypocalcemia was detected two weeks after the first denosumab administration, during routine biochemical evaluation. The patient reported only a mild nonspecific paresthesia after medical questioning, without relevant clinical symptoms. Despite the severity of the hypocalcemia, serum calcium levels began to improve after a short period of low-dose calcium and calcitriol therapy, though complete stabilization and normalization occurred after several weeks.

**Conclusion:**

This case highlights the importance to consider severe paucisymptomatic or asymptomatic hypocalcemia as a possible side effect in bone-metastatic patients treated with denosumab. It is advisable to monitor serum calcium levels even in the absence of typical hypocalcemia-related symptoms.

## 1 INTRODUCTION

Denosumab is a fully-humanized monoclonal neutralizing antibody inhibiting the activation of the RANK/RANKL/OPG signaling pathway through competitive binding with RANKL [1]. Its primary effect is the inhibition of osteoclast-mediated bone resorption and bone loss, making it widely used in the treatment of metabolic bone diseases (including postmenopausal osteoporosis, male osteoporosis, and glucocorticoid-induced osteoporosis) [2]. Notably, preclinical models and clinical studies have shown denosumab to have both direct and indirect anti-tumor effects, leading to its adoption as a standard treatment for patients with bone metastases [3] as well as in the management of cancer treatment-induced bone loss [4]. The therapeutic benefits of denosumab administration (120 mg monthly) are mainly related to its suppressive effect on bone remodeling, which is higher than that observed for zoledronic acid (4 mg monthly) [5]. However, the use of denosumab in cancer patients is burdened by the risk of all grades of hypocalcemia. Some Authors reported that, unlike denosumab, bisphosphonates do not significantly affect serum calcium levels, exhibiting a better safety profile regarding hypocalcemia [6]. This is primarily because RANKL inhibitors are more potent in reducing bone turnover and, consequently, calcium release into circulation [7]. Hypocalcemia following denosumab administration has been particularly noted in patients with bone metastases, especially from breast and prostate cancer, making serum calcium monitoring essential for appropriate management. Herein, we report a case of severe hypocalcemia following denosumab administration in a patient with sigmoid cancer and multiple metastases, including bone involvement.

## 2 CASE PRESENTATION

A 78-year-old man came to our attention in January 2023 with a diagnosis of sigmoid adenocarcinoma with liver and bone metastasis. His previous medical history did not reveal important diseases. He had mild hypertension on adequate medical therapy (Olmesartan 10 mg/day). Imaging studies, including CT scan and bone scintigraphy, revealed radiodense bone lesions in the clivus, in the right mastoid bone, in the right sternoclavicular joint, as well as multiple osteoblastic spinal and rib metastases. A 2 cm hepatic lesion, suggestive of metastasis, and a 3 cm paravesical metastatic lymph node had also been reported. Biopsy of the sigmoid colon showed a neoplasia expressing keratin AE1/AE3 positivity, that suggested poorly differentiated adenocarcinoma (BerEP4+; CD56 -; p40 -; WT1. Immunophenotype: MLH1+; PMs2+; MSH2+; MSH6+). The patient underwent proctoscopy and palliative metallic stent placement. Genetic testing for DPYD revealed a wild-type phenotype. In February 2023, the patient started chemotherapy with leucovorin calcium (folinic acid), fluorouracil, and oxaliplatin (FOLFOX regimen, 12 cycles), while in April 2023, he started radiotherapy targeted on vertebral bone metastases (lumbar metamers). In September 2023, panitumumab, combined with fluorouracil and folinic acid, was started as maintenance therapy. Follow-up bone scintigraphy in November 2023 showed disease progression, reporting an increased uptake in the sternoclavicular joint, as well as in cervical and dorsal metamers, while the uptake was reduced in lumbar metamers. An 18-FDG PET confirmed extensive bone involvement and pathological uptake in the known paravescical lymph node, while the liver uptake was less intense. In January 2024, the patient started therapy with subcutaneous denosumab 120 mg/month. Serum calcium levels were normal before the first denosumab administration (9.5 mg/dl). Vitamin D (25-hydroxycholecalciferol) levels were normal (39 ng/ml) under cholecalciferol supplementation (1.000 UI/day) that had been initiated four months earlier (baseline vitamin D: 19 ng/ml). PTH levels were mildly elevated before cholecalciferol supplementation (72 pg/ml, n.v. 10-65), with normal magnesium levels (2.01 mg/dl). Two weeks after denosumab administration, routine biochemical tests revealed severe hypocalcemia (serum calcium 6.2 mg/dl (1.55 mmol/L) (Table **1**), with normal serum albumin levels and secondary hyperparathyroidism (PTH 332 pg/ml). Biochemical evaluations are reported in Table (**1**). An electrocardiogram was performed, showing normal parameters. Thyroid ultrasound showed a small hypoechoic 8 mm nodule without ultrasonographic suspicious features. No enlarged parathyroid glands were reported, and cervical lymph nodes displaying normal shape and hyperechoic hilum were described.

**Fig. (1):**
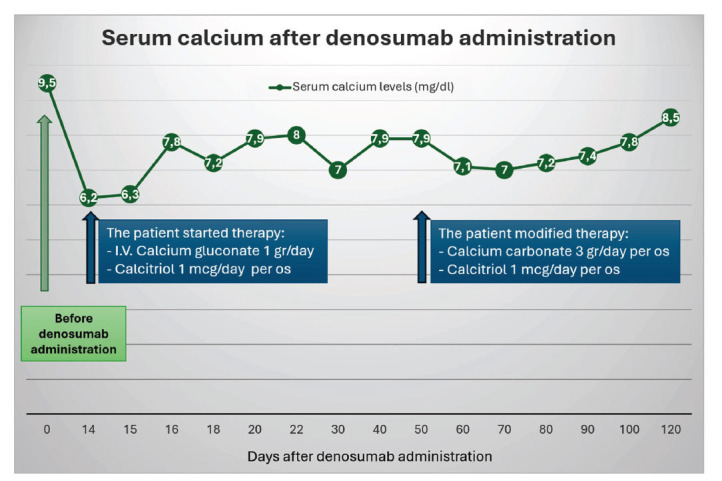
Serum calcium monitoring after denosumab administration.

Despite the severity of hypocalcemia, the patient was asymptomatic. Only after medical evaluation and questioning, the patient revealed mild paresthesias. Calcitriol 1 mcg/day and calcium gluconate (1 gr/10 ml i.v./day) were started, with close monitoring of serum calcium levels (Fig. **1**). After four weeks of treatment, serum calcium levels improved, allowing a transition from i.v. calcium gluconate to oral calcium carbonate (3 gr/day). Following a transient phase of mild asymptomatic hypocalcemia, serum calcium levels normalized with this therapeutic scheme in about 4 months after denosumab administration. Denosumab therapy was discontinued after the first administration. Calcium carbonate and calcitriol were gradually tapered and stopped, with serum calcium levels remaining normal.

## 3 DISCUSSION

This case highlights the rare occurrence of severe asymptomatic hypocalcemia following denosumab administration in a patient with sigmoid cancer and bone metastases. Bone metastases are detected in about 5% of cancer patients at diagnosis, with breast and prostate cancer (65-75%), lung and bladder cancer (30-40%) and renal cancer (20-35%) being the most common primary tumors [8]. Bone metastases from cancers of the digestive system, as in the reported patient, are much less common (5%). The use of denosumab in patients with bone metastases is based on its ability to inhibit RANKL, thereby reducing bone resorption and decreasing the incidence of skeletal-related events [9]. However, due to the mechanism of action, the use of denosumab can also lead to hypocalcemia. If mild hypocalcemia, defined as serum calcium (adjusted for albumin) between 7.5 and 8.5 mg/dl, accounts for up to 7% of patients treated with denosumab for osteoporosis [10], the incidence is higher in patients with bone metastases. Huynh and Coll reported an incidence of 14% of denosumab-associated hypocalcemia in patients with osteoporosis or metastatic bone disease within 6 months of treatment and despite widespread use of appropriate calcium/cholecalciferol supplementation [11]. Severe hypocalcemia (serum calcium < 7 mg/dl) is rarer, affecting less than 3% of patients. In our patient, severe hypocalcemia occurred despite adequate vitamin D levels under cholecalciferol supplementation. Serum calcium levels at baseline were normal, so dietary calcium intake appeared to be adequate, and no calcium supplementation had been proposed. We also analyzed a possible mechanism, induced by previous chemotherapy, for the occurrence of hypocalcemia. Oxaliplatin and 5-Fluorouracil, which are part of the combination FOLFOX-Panitumumab, are rarely responsible for electrolyte disturbances, including hypomagnesemia and hypocalcemia [12]. Regarding the anti-epidermal growth factor receptor (EGFR) monoclonal antibody panitumumab, electrolyte abnormalities, including hypomagnesemia, are reported among the characteristic side effects of anti-EGFR antibodies, while hypocalcemia is rarely reported [13]. Both the electrolyte abnormalities are suggested to be due to the same mechanisms, being linked to inhibition of EGFR-mediated magnesium re-absorption from urine in renal tubules and inhibition of EGFR-mediated magnesium absorption in the intestinal tract [14]. Notably, magnesium regulates blood calcium levels *via* its control of PTH secretion, and calcium plays a key role in regulating PTH secretion [15]. In detail, a deficiency in intracellular magnesium suppresses PTH secretion, potentially leading to a decrease in serum PTH and calcium levels. Therefore, patients treated with anti-EGFR antibodies are at risk of developing hypomagnesemia and concurrent hypocalcemia [15]. However, blood calcium and magnesium in our patient assessed after the end of the FOLFOX-panitumumab combination and before starting denosumab were within the normal ranges, respectively. Nevertheless, as the majority of the body's magnesium is in bone, while only 1% is in the blood, the body's magnesium may be deficient even if the serum magnesium level is within the normal range. Undetected hypomagnesemia could be the underlying mechanism of the reported denosumab-induced hypocalcemia, following the first-line treatment of metastatic colon cancer. Unfortunately, tools are currently not available to demonstrate the suggested “masked hypomagnesemia” as a potential mechanism of denosumab-related hypocalcemia diagnosed in the reported case. Moreover, during denosumab therapy, both osteolytic and osteoblastic bone metastases can cause hypocalcemia like the “hungry bone syndrome”, occurring more frequently in patients with severe hyperparathyroidism after surgical treatment and described after parathyroidectomy or kidney transplantation [16, 17], as a result of excessive calcium uptake in the bone [18]. Denosumab administration is also associated with a transient increase of 1,25(OH)_2_D_3_ and PTH at one week, followed by a progressive decrease [19]. In our patient, a significant rise in PTH levels was observed, likely as a compensatory response to severe hypocalcemia. Specific risk factors for denosumab-induced hypocalcemia have been reported, including prostate cancer, small-cell lung cancer, multiple osteoblastic bone metastases, renal dysfunction, and elevated bone-specific alkaline phosphatase levels [20, 21]. Renal dysfunction also represents a risk factor for the development of hypocalcemia in patients with solid tumors and bone metastases [22]. Aside from elevated alkaline phosphatase levels, no other risk factors were present in our patient. The clinical experience of denosumab-induced hypocalcemia in bone-metastatic colorectal cancer is limited, with most studies reporting this side effect being based on patients with breast and prostate cancers [23]. Therefore, this case represents a rare report of this side effect in a patient with sigmoid cancer. Moreover, while hypocalcemia typically presents with well-known clinical manifestations [23], in our patient, the hypocalcemia was severe but clinically asymptomatic. Electrocardiographic findings were normal, and no neurological manifestations were reported. Indeed, hypocalcemia was discovered during routine biochemical evaluation. The mechanisms of denosumab-induced asymptomatic hypocalcemia have not been reported yet. It is not possible to exclude that older age can reduce the perception of classical symptoms associated with hypocalcemia. Other case reports described severe asymptomatic hypocalcemia in older patients [24, 25]. Another possible explanation can be related to chemotherapy-induced neuropathy, which can cause chronic neuropathic pain, masking clinical symptoms of acute hypocalcemia. Nevertheless, severe hypocalcemia can represent a life-threatening condition due to cardiological and neurological complications. Therefore, management of denosumab-induced hypocalcemia requires prompt recognition, correction of serum calcium levels, and prevention of recurrent episodes. In cases of severe hypocalcemia, such as in our patient, intravenous calcium may be initially required, followed by oral calcium and an active form of vitamin D. Interestingly, despite the severity of hypocalcemia, our patient’s serum calcium levels improved with relatively low doses of calcium gluconate (1 gr/day) and calcitriol (1 mcg/day), compared to higher doses therapeutic schemes proposed in literature to treat severe hypocalcemia [23, 26]. As reported in literature, serum calcium levels normalized after several weeks (4 months) of treatment. Denosumab-induced hypocalcemia is generally reversible upon discontinuation of therapy, but it is important to underline that denosumab has a prolonged duration of action lasting up to six months, necessitating ongoing biochemical monitoring. As a general protocol, which should be tailored to each patient, we monitor serum calcium levels in patients with bone metastases receiving 120 mg of denosumab monthly at 2 weeks, 6 weeks, and 12 weeks, and then every 3 to 6 months, depending on clinical progression. Prior to starting treatment, we also advise patients to promptly report any potential symptoms of hypocalcemia. In our case, serum calcium levels have remained normal and therapy with calcium and calcitriol has been discontinued.

## CONCLUSION

In conclusion, our case report highlights a particular and rare case of denosumab-related severe hypocalcemia in a patient with bone metastases from colorectal cancer. The peculiar factor is that severe hypocalcemia was asymptomatic and detected during routine biochemical evaluation. This underscores the importance of routine biochemical monitoring in patients receiving denosumab, even in the absence of signs/symptoms of hypocalcemia. The rapid normalization of serum calcium with low-dose therapy also emphasizes the value of early detection and intervention.

## Figures and Tables

**Table 1:** Biochemical evaluation performed 2 weeks after denosumab administration.

Biochemical Data	Levels	Normal Reference Range
Glycemia	103 mg/dl	70-100
Creatinine	0.97 mg/dl	9.5 - 1.3
**Calcium**	**6.2 mg/dl**	**8.4 - 10.4**
**Phosphorus**	**1.65 mg/dl**	**2.5 - 4.5**
Magnesium	1.73 mg/dl	1.5 - 2.5
Total Bilirubin	0.42 mg/dl	< 1.4
Indirect bilirubin	0.23 mg/dl	< 0.3
Alanine aminotransferase	14 U/L	< 40
Aspartate aminotransferase	10 U/L	< 41
Sodium	140 mEq/L	135-153
Potassium	5 mEq/L	3.5 - 5.3
**Alkaline phosphatase**	**464 U/L**	**40-130**
Urinary calcium	10 mg/24 h	100-300
Albumin	4 gr/dl	3.6 - 5.2
**PTH**	**332 pg/ml**	**10-65**

## Data Availability

All data generated or analysed during this study are included in this published article.

## LIST OF ABBREVIATIONS

EGFR: Epidermal Growth Factor Receptor
FA: Folinic Acid

## STANDARDS OF REPORTING

CARE guidelines were followed.
